# Supplementary material for: Effects of Incorporation of Porous Tapioca Starch on the Quality of White Salted (Udon) Noodles
Source: Foods. 2023 Apr 16;12(8):1662. doi: 10.3390/foods12081662 (PMC10137948; doi:10.3390/foods12081662)
Supplement: Supplementary file 1 [file foods-12-01662-s001.zip › foods-2311906-supplementary.pdf]

Table S1 Cooking quality and textural attributes of noodles

Wheat variety	% Porous starch added	Sample ID	Optimal cooking time (min)	Cooking loss (%)	Water absorption (%)	Texture, g Force	Turbidity (Au)
Commercial	0	C0	11.10 ±0.10	3.695± 0.005	119.39 ± 0.10	2952 ±17.50	0.35 ±0.03
	5	C5	10.60± 0.16	3.7± 0.08	101.34 ±0.07	3494 ±5.77	0.38± 0.01
	10	C10	10.35 ±0.12	4.55± 0.04	131.03 ±0.06	2646.5±120.51	0.5± 0.01
Mace	0	M0	9.35 ±0.05	4.6± 0.10	94.65 ±0.09	4180±150.13	0.42± 0.01
	5	M5	8.4 ±0.10	4.7±0.10	96.17 ±0.92	3707 ±57.73	0.49± 0.01
	10	M10	8 ± 0.00	4.95±0.10	100.47 ±3.35	2678 ±56.01	0.51 ±0.01
Zen	0	Z0	10.25± 0.25	4.55 ±0.05	100.38 ±0.14	3840.67 ±103.94	0.43± 0.01
	5	Z5	7.35 ±0.15	4.86± 0.35	80.52 ±0.08	4647 ±85.73	0.46 ±0.01
	10	Z10	7.05 ±0.05	4.97±0.35	82.71 ±0.12	4043.33 ±330.44	0.56 ±0.01
Kinsei	0	K0	9.11 ±0.01	2.8 ±0.10	86.3 ±0.06	3739 ±180.14	0.37± 0.01
	5	K5	7.45± 0.05	4.745 ±0.04	98.76 ±0.43	2943.67 ±168.30	0.5 ±0.01
	10	K10	7.27 ±0.07	4.96± 0.05	97.68 ±0.46	2409.33± 92.76	0.54± 0.01
Ninja	0	N0	10.425 ±0.02	4.315±0.11	99.77 ±0.23	4318.33 ±95.885	0.4 ±0.01
	5	N5	8.09 ±0.09	4.63 ±0.07	86.49± 1.14	2496 ±334.31	0.48± 0.01
	10	N10	8.05 ±0.05	4.69± 0.09	91.82 ±1.60	2876.33± 73.87	0.52± 0.23

Table S2 Pairwise comparisons of different quality attributes of udon noodles among wheat varieties

Factors			OCT		WAC		Cooking loss		Turbidity		Texture	
PS	(I) Variety	(J) Variety	Mean Diff (I-J)	P value	Mean Diff (I-J)	P value	Mean Diff (I-J)	P value	Mean Diff (I-J)	P value	Mean Diff (I-J)	P value
0	Com	Kinsei	1.985*	<.001	33.095*	<.001	.895*	<.001	-.020	.123	-592.5*	.047
		Mace	1.750*	<.001	24.745*	<.001	-.905*	<.001	-.070*	<.001	-1141.0*	<.001
		Ninja	.675*	<.001	19.625*	<.001	-.620*	<.001	-.050*	<.001	-1332.0*	<.001
		Zen	.850*	<.001	19.010*	<.001	-.860*	<.001	-.085*	<.001	-962.0*	.003
	Kinsei	Com	-1.985*	<.001	-33.095*	<.001	-.895*	<.001	.020	.123	592.5*	.047
		Mace	-.235	.131	-8.350*	<.001	-1.800*	<.001	-.050*	<.001	-548.5	.063
		Ninja	-1.310*	<.001	-13.470*	<.001	-1.515*	<.001	-.030*	.027	-739.5*	.016
		Zen	-1.135*	<.001	-14.085*	<.001	-1.755*	<.001	-.065*	<.001	-369.5	.197
	Mace	Com	-1.750*	<.001	-24.745*	<.001	.905*	<.001	.070*	<.001	1141.0*	<.001
		Kinsei	.235	.131	8.350*	<.001	1.800*	<.001	.050*	<.001	548.5	.063
		Ninja	-1.075*	<.001	-5.120*	.004	.285*	.027	.020	.123	-191.0	.495
		Zen	-.900*	<.001	-5.735*	.002	.045	.704	-.015	.240	179.0	.523
	Ninja	Com	-.675*	<.001	-19.625*	<.001	.620*	<.001	.050*	<.001	1332.0*	<.001
		Kinsei	1.310*	<.001	13.470*	<.001	1.515*	<.001	.030*	.027	739.5*	.016
		Mace	1.075*	<.001	5.120*	.004	-.285*	.027	-.020	.123	191.0	.495
		Zen	.175	.252	-.615	.684	-.240	.057	-.035*	.012	370.0	.196
	Zen	Com	-.850*	<.001	-19.010*	<.001	.860*	<.001	.085*	<.001	962.0*	.003
		Kinsei	1.135*	<.001	14.085*	<.001	1.755*	<.001	.065*	<.001	369.5	.197
		Mace	.900*	<.001	5.735*	.002	-.045	.704	.015	.240	-179.0	.523
		Ninja	-.175	.252	.615	.684	.240	.057	.035*	.012	-370.0	.196
5	Com	Kinsei	3.150*	<.001	2.575	.103	-1.045*	<.001	-.120*	<.001	584.5*	.049
		Mace	2.200*	<.001	5.165*	.003	-1.000*	<.001	-.110*	<.001	-268.0	.342
		Ninja	2.510*	<.001	14.850*	<.001	-.930*	<.001	-.100*	<.001	1259.0*	<.001
		Zen	3.250*	<.001	20.815*	<.001	-1.165*	<.001	-.080*	<.001	-1084.0*	.001
	Kinsei	Com	-3.150*	<.001	-2.575	.103	1.045*	<.001	.120*	<.001	-584.5*	.049
		Mace	-.950*	<.001	2.590	.101	.045	.704	.010	.427	-852.5*	.007
		Ninja	-.640*	<.001	12.275*	<.001	.115	.338	.020	.123	674.5*	.026
		Zen	.100	.507	18.240*	<.001	-.120	.318	.040*	.005	-1668.5*	<.001
	Mace	Com	-2.200*	<.001	-5.165*	.003	1.000*	<.001	.110*	<.001	268.0	.342
		Kinsei	.950*	<.001	-2.590	.101	-.045	.704	-.010	.427	852.5*	.007
		Ninja	.310	.052	9.685*	<.001	.070	.556	.010	.427	1527.0*	<.001
		Zen	1.050*	<.001	15.650*	<.001	-.165	.176	.030*	.027	-816.0*	.009
	Ninja	Com	-2.510*	<.001	-14.850*	<.001	.930*	<.001	.100*	<.001	-1259.0*	<.001
		Kinsei	.640*	<.001	-12.275*	<.001	-.115	.338	-.020	.123	-674.5*	.026
		Mace	-.310	.052	-9.685*	<.001	-.070	.556	-.010	.427	-1527.0*	<.001
		Zen	.740*	<.001	5.965*	.001	-.235	.061	.020	.123	-2343.0*	<.001
	Zen	Com	-3.250*	<.001	-20.815*	<.001	1.165*	<.001	.080*	<.001	1084.0*	.001
		Kinsei	-.100	.507	-18.240*	<.001	.120	.318	-.040*	.005	1668.5*	<.001
		Mace	-1.050*	<.001	-15.650*	<.001	.165	.176	-.030*	.027	816.0*	.009
		Ninja	-.740*	<.001	-5.965*	.001	.235	.061	-.020	.123	2343.0*	<.001
10	Com	Kinsei	3.075*	<.001	33.350*	<.001	-.410*	.003	-.010	.427	171.25	.540

	Mace	2.350*	<.001	30.565*	<.001	-.400*	.004	.020	.123	-71.75	.797
	Ninja	2.300*	<.001	39.215*	<.001	-.140	.247	.010	.427	-282.25	.318
	Zen	3.300*	<.001	48.325*	<.001	-.425*	.002	-.030*	.027	-1596.75*	<.001
Kinsei	Com	-3.075*	<.001	-33.350*	<.001	.410*	.003	.010	.427	-171.25	.540
	Mace	-.725*	<.001	-2.785	.080	.010	.933	.030*	.027	-243.0	.388
	Ninja	-.775*	<.001	5.865*	.001	.270*	.035	.020	.123	-453.5	.118
	Zen	.225	.147	14.975*	<.001	-.015	.899	-.020	.123	-1768.0*	<.001
Mace	Com	-2.350*	<.001	-30.565*	<.001	.400*	.004	-.020	.123	71.75	.797
	Kinsei	.725*	<.001	2.785	.080	-.010	.933	-.030*	.027	243.0	.388
	Ninja	-.050	.738	8.650*	<.001	.260*	.041	-.010	.427	-210.5	.453
	Zen	.950*	<.001	17.760*	<.001	-.025	.833	-.050*	<.001	-1525.0*	<.001
Ninja	Com	-2.300*	<.001	-39.215*	<.001	.140	.247	-.010	.427	282.25	.318
	Kinsei	.775*	<.001	-5.865*	.001	-.270*	.035	-.020	.123	453.5	.118
	Mace	.050	.738	-8.650*	<.001	-.260*	.041	.010	.427	210.5	.453
	Zen	1.000*	<.001	9.110*	<.001	-.285*	.027	-.040*	.005	-1314.5*	<.001
Zen	Com	-3.300*	<.001	-48.325*	<.001	.425*	.002	.030*	.027	1596.75*	<.001
	Kinsei	-.225	.147	-14.975*	<.001	.015	.899	.020	.123	1768.0*	<.001
	Mace	-.950*	<.001	-17.760*	<.001	.025	.833	.050*	<.001	1525.0*	<.001
	Ninja	-1.000*	<.001	-9.110*	<.001	.285*	.027	.040*	.005	1314.5*	<.001

Table S3 Pairwise comparisons of different quality attributes of udon noodles among added porous starch

Factors			OCT		WAC		Cooking loss		Turbidity		Texture	
**Variety**	**(I) PS**	**(J) PS**	**Mean Diff (I-J)**	**P value**	**Mean Diff (I-J)**	**P value**	**Mean Diff (I-J)**	**P value**	**Mean Diff (I-J)**	**P value**	**Mean Diff (I-J)**	**P value**
Com	0	5	.500*	.004	18.055*	<.001	-.005	.966	-.030*	.027	-519.5	.077
		10	.750*	<.001	-11.640*	<.001	-.855*	<.001	-.180*	<.001	314.75	.268
	5	0	-.500*	.004	-18.055*	<.001	.005	.966	.030*	.027	519.5	.077
		10	.250	.110	-29.695*	<.001	-.850*	<.001	-.150*	<.001	834.25*	.008
	10	0	-.750*	<.001	11.640*	<.001	.855*	<.001	.180*	<.001	-314.75	.268
		5	-.250	.110	29.695*	<.001	.850*	<.001	.150*	<.001	-834.25*	.008
Kinsei	0	5	1.665*	<.001	-12.465*	<.001	-1.945*	<.001	-.130*	<.001	657.5*	.030
		10	1.840*	<.001	-11.385*	<.001	-2.160*	<.001	-.170*	<.001	1078.5*	.001
	5	0	-1.665*	<.001	12.465*	<.001	1.945*	<.001	.130*	<.001	-657.5*	.030
		10	.175	.252	1.080	.477	-.215	.084	-.040*	.005	421.0	.144
	10	0	-1.840*	<.001	11.385*	<.001	2.160*	<.001	.170*	<.001	-1078.5*	.001
		5	-.175	.252	-1.080	.477	.215	.084	.040*	.005	-421.0	.144
Mace	0	5	.950*	<.001	-1.525	.320	-.100	.403	-.070*	<.001	353.5	.216
		10	1.350*	<.001	-5.820*	.001	-.350*	.009	-.090*	<.001	1384.0*	<.001
	5	0	-.950*	<.001	1.525	.320	.100	.403	.070*	<.001	-353.5	.216
		10	.400*	.016	-4.295*	.011	-.250*	.048	-.020	.123	1030.5*	.002
	10	0	-1.350*	<.001	5.820*	.001	.350*	.009	.090*	<.001	-1384.0*	<.001
		5	-.400*	.016	4.295*	.011	.250*	.048	.020	.123	-1030.5*	.002
Ninja	0	5	2.335*	<.001	13.280*	<.001	-.315*	.016	-.080*	<.001	2071.5*	<.001
		10	2.375*	<.001	7.950*	<.001	-.375*	.006	-.120*	<.001	1364.5*	<.001
	5	0	-2.335*	<.001	-13.280*	<.001	.315*	.016	.080*	<.001	-2071.5*	<.001
		10	.040	.789	-5.330*	.003	-.060	.613	-.040*	.005	-707.0*	.021
	10	0	-2.375*	<.001	-7.950*	<.001	.375*	.006	.120*	<.001	-1364.5*	<.001
		5	-.040	.789	5.330*	.003	.060	.613	.040*	.005	707.0*	.021
Zen	0	5	2.900*	<.001	19.860*	<.001	-.310*	.018	-.025	.059	-641.5*	.033
		10	3.200*	<.001	17.675*	<.001	-.420*	.003	-.125*	<.001	-320.0	.260
	5	0	-2.900*	<.001	-19.860*	<.001	.310*	.018	.025	.059	641.5*	.033
		10	.300	.059	-2.185	.161	-.110	.359	-.100*	<.001	321.5	.258
	10	0	-3.200*	<.001	-17.675*	<.001	.420*	.003	.125*	<.001	320.0	.260
		5	-.300	.059	2.185	.161	.110	.359	.100*	<.001	-321.5	.258

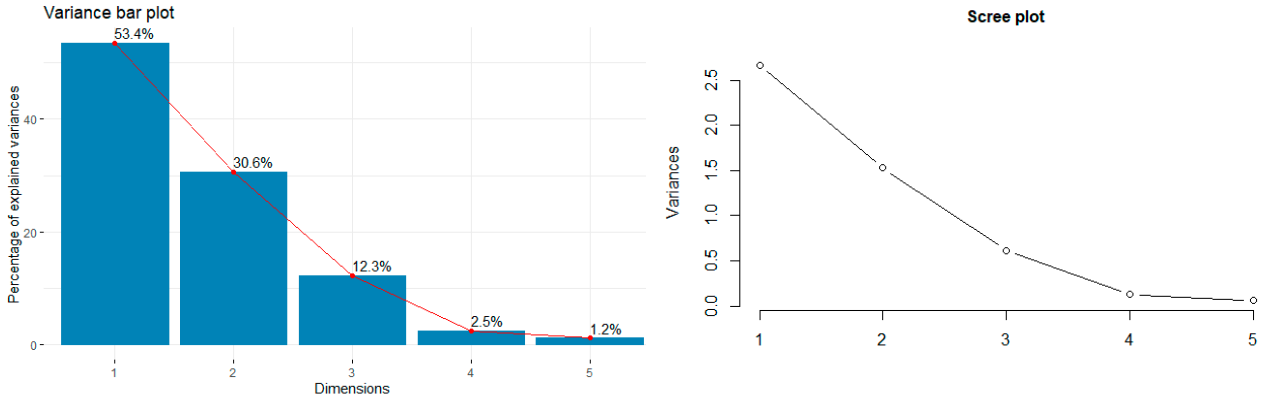

Figure S1. Variance bar plot and screen plot showing percentage of explained variances and eigen values of the principal components.

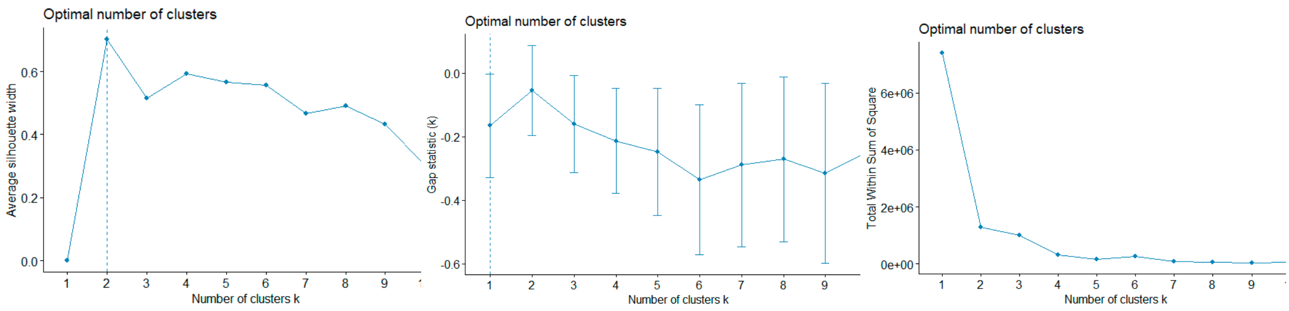

Figure S2. Results of optimum number of clusters as obtained from Silhouette, Gap statistic, and Elbow methods.
